# Effect of neuromuscular electrical stimulation in glycogen muscle reserves because of ingestion of ethanol: a study in rats

**DOI:** 10.1590/S1679-45082013000100015

**Published:** 2013

**Authors:** Ederson Luís Limoni, Eder João de Arruda

**Affiliations:** 1 Anhanguera Educacional, Santa Bárbara do Oeste, SP, Brazil

**Keywords:** Rats, Glycogen/metabolism, Electric stimulation, Ethanol/adverse effects

## Abstract

**Objective:**

To evaluate the effects of alcoholic ingestion and neurostimulation on the muscle glycogen reserve, body weight, blood sugar, and weight of the soleus muscle.

**Methods:**

Twenty male rats were distributed into four experimental groups (n=5), namely, Control, Ethanol, Electrostimulated, and Ethanol+Electrostimulated. The study lasted for 22 days. The groups submitted to the use of ethanol received the substance diluted in water, which was consumed during the entire experimental period. The groups that received electrostimulation, undersedationfor the procedure, had their left hind leg shaved, and the current was applied daily for 7 days, in 20-minute sessions. Next, after induced alcoholism and electrical stimulation in the corresponding groups, the animals were euthanized so that their muscles could be sent for glycogen analysis.

**Results:**

The Ethanol group displayed a lower body weight when compared to the Control and Electrostimulated groups; the Ethanol+Electrostimulated groups had a lower body weight compared to the Control and Electrostimulated groups, but were in a better situation when compared to the Ethanol group. As to glycogen capture, it was noted that the Ethanol group demonstrated resistance to blood glucose capture, whereas the Ethanol Electrostimulated group showed better capture than the other groups. As to muscle weight, it was observed that the Ethanol group had a lower weight than did the Controls, and that the Electrostimulated group weight greater when compared to the Control and Ethanol groups, respectively. On the other hand, the Ethanol+Electrostimulated groups showed no significant difference relative to the Controls, but had better results when compared to the Ethanol group.

**Conclusion:**

Chronic exposure to alcohol showed a direct relationship with reduced muscle and body weight, and in glycogen capture and muscle reserves, besides favoring innumerous organic disorders, thus interfering in rehabilitation processes.

## RESUMO

**Objetivo:**

Avaliar as ações da ingestão alcoólica e da neuroestimulação sobre a reserva de glicogênio muscular, o peso corporal, a glicemia sanguínea e o peso do músculo sóleo.

**Métodos:**

Vinte ratos machos foram distribuídos em quatro grupos experimentais (n=5), a saber, Controle, Etanol, Eletroestimulado e Etanol+Eletroestimulado. A pesquisa durou 22 dias. Os grupos submetidos ao uso do etanol receberam a substância diluída em água, que foi consumida durante todo o período experimental. Os grupos que receberam a eletroestimulação, sob sedação para o procedimento, tiveram o membro posterior esquerdo tricotomizado, de forma que a aplicação da corrente foi realizada diariamente, por 7 dias, em sessões de 20 minutos. Na sequência, após a indução ao alcoolismo e a aplicação da estimulação elétrica nos correspondentes grupos, os animais foram eutanasiados, para que os músculos fossem encaminhados para análise do glicogênio.

**Resultados:**

O Grupo Etanol apresentou menor peso corporal quando comparado ao Controle e ao Eletroestimulado; o Grupo Etanol+Eletroestimulado apresentou peso menor quando comparado aos Grupos Controle e Eletroestimulado, mas esteve em melhor situação ao se comparar ao Grupo Etanol. Quanto à capitação glicogênica, notou-se que o Grupo Etanol demonstrou quadro de resistência à capitação da glicose sanguínea, sendo que o etanol eletroestimulado demonstrou melhor captação que os demais grupos. Em relação ao peso da musculatura, pode-se observar que o Grupo Etanol apresentou menor peso que o Controle e que o Grupo Eletroestimulado apresentou aumento da pesagem ao compararse aos grupos Controle e Etanol, respectivamente. Já o Grupo Etanol+Eletroestimulado não apresentou diferença significativa em relação ao Controle, mas teve melhores resultados ao contrapesar com o Grupo Etanol.

**Conclusão:**

A exposição crônica ao álcool apresentou relação direta com a redução do peso do muscular e corpóreo, e da captação de glicogênio e de suas reservas musculares, além de favorecer inúmeras desordens orgânicas, podendo, assim, interferir em processos de reabilitação.

## INTRODUCTION

The ingestion of ethyl alcohol (ethanol) may set off pathological metabolic changes in the different systems of the human body. In this sense, there are alterations in functions of the nervous system, glucose metabolism, lipids and proteins, and particularly, the nutritional aspects of organs such as the liver, pancreas, stomach, and intestines^(1)^.

Based on clinical and histological tests of the muscles, studies have demonstrated that chronic alcoholics show different degrees of muscle weakness, which can evolve to an acute condition of alcoholic myopathy. Muscle atrophy, type-grouping formation, proliferation, and in parallel, mitochondrial accumulation are serious alterations that deserve attention^(2,3)^.

With the intention of minimizing the events triggered by deleterious conditions that act on muscle tissue, various techniques have been proposed by physical therapy, and electric neuromuscular stimulation (EENM) has shown good results in maintaining muscle conditions or in retarding catabolism^(3)^.

Minimization of peripheral nerve utility due to pathological events resulting in a deficit of contractility culminates in severe atrophy, which may be irreversible because of the intense proliferation of connective tissue as a result of the inflammatory process. In this way, EENM has also been proven effective in promoting an elevation in the contractile activity of muscle fibers and in improving the dynamics of glucose capture and metabolism, with proof of an increase in cellular metabolic routes^(4)^.

With an emphasis on carbohydrate metabolism, the exaggerated consumption of ethanol shows a direct relationship with increased risk factors for cardiovascular diseases and the development of type 2 diabetes mellitus^(5)^.

Along this line of investigation, literature is scarce and does not detail aspects of rehabilitation, a fact that needs to be highlighted on the part of physical therapy, because of the wide range of resources available for intervention in organic systems that are in deleterious conditions due to chronic use of large quantities of ethanol^(4)^.

In the meantime, we presume that in the context of rehabilitation by electrostimulation there may be positive results, since literature shows positive results with the application of the resource^(3)^. Nevertheless, ethanol ingestion may interfere directly in the responses of muscle and hepatic tissue with the use of therapeutic resources. Thus, the present project had the purpose of investigating the effectiveness of EE in the condition of ethanol ingestion.

## OBJECTIVE

To evaluate the effects of alcohol ingestion and neurostimulation on muscle glycogen reserve, body weight, blood sugar, and soleus muscle weight.

## METHODS

The study was approved by the Anhanguera Educacional Ltda. Committee of Ethics on the Use of Animals (CEUA/AESA), #1.025. It was carried out from January to October, 2011.

Twenty Wistar male rats were used, with 3 to 4 months of age and 200 to 300g in weight. They were maintained under controlled conditions of the animal house (temperature of 23±2°C and 12-hour light/dark photoperiodic cycle). The practical period with the animals in the study lasted 22 days, from induction to myopathy under the use of 4% ethanol solution, with free access to the groups treated with ethanol as well as the intervention of functional electrical stimulation (FES) with electrodes applied one at the origin and the other at the insertion of the triceps surae muscle in the groups treated with EE. Anteceding the experimental period, the animals remained for 48 hours in adaptation to the conditions of the research animal house.

### Experimental groups

The animals were divided into four experimental groups (n=5; Control group – C, Ethanol-treated group – Et, Electrostimulated group – EE, and the group treated with Ethanol and Electrostimulation – EtEE).

### Ethanol treatment

The groups treated with ethanol received a 4% solution of ethanol, with free access for 14 days. The ethanol solution was prepared at least 20 hours ahead of the time of animal availability^(4)^.

### EENM exposure

After the alcoholic ingestion period, the animals submitted to EE were sedated, and the left hind leg was shaved for greater effectiveness of the EE. The EE was performed daily for 7 days, in 20-minute sessions. The electrodes were placed one at the origin and the other at the insertion of the triceps surae muscle, as per the established protocol.

The equipment used for EE was Dualpex 961 (Quark^^®^^), besides four silicon-carbon electrodes with 1cm^2^ each and transducer gel.

As current parameters, a frequency of 10Hz was established and a phase width of 0.4ms. Current intensity was standardized at 5.0mA, and every 5 minutes, 1.0mA was added to avoid accommodation.

### Blood glucose analysis

Blood glucose was evaluated by means of a glucose tolerance test (GTT), with blood sampling for analysis at 0, 10, 20, 30, and 60 minutes after electrostimulation in both groups.

### Weighing the muscle

The soleus muscle (S) was removed in one piece, from the tendon to its insertion. Next, it was weighed (wet weight) on analytical scales.

### Anesthesia and euthanasia procedures

On the 15^th^ day, after induced alcoholism, the electrostimulation procedure was initiated. The animals were sedated by intraperitoneal (ip) route, bearing in mind the discomfort of the intravenous route, with the use of ketamine (70mg/kg) and xylazine (10mg/kg), that afford hypnotic effects and muscular relaxation for up to 40 minutes. On the other hand, euthanasia was performed after the 22^nd^ day after treatment with EE in the groups that received it, using sodium pentobarbital anesthetic (100mg/kg) by intravenous route (caudal vein), as per ethical and practical principles of use of experimental animals.

### Determination of muscle glycogen

At the end of the study, after euthanasia, samples of the soleus, and white and red gastrocnemius muscles were removed and digested in hot 30% KOH. The glycogen precipitated after the use of ethanol. Between the precipitation phases, the sample was centrifuged at 3.000rpm for 15 minutes^(6)^. Values were expressed in mg/100mg of wet weight.

### Statistical analysis

The BioEstat 5.0^^®^^ program was used, with the data submitted to the normality test (Kolmogorov-Smirnov), with a critical level set at 5% (p<0.05).

## RESULTS

**Figure 1:**
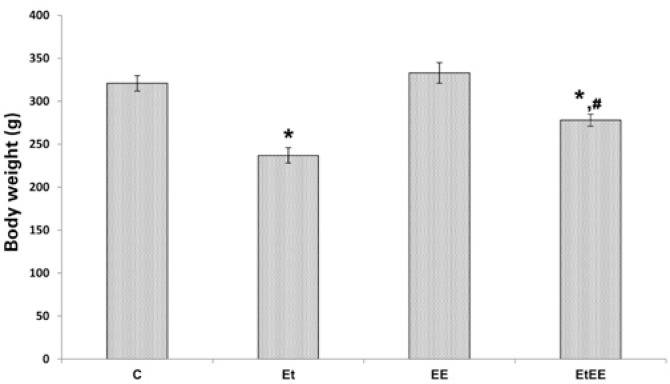
Graph of body weight of the Groups Control (C), Ethanol (Et), Electrostimulated (EE) and e Ethanol+Electrostimulated (EtEE). The values show mean±SEM, as p as significance value.

Initially, the body weight of the animals in the groups was evaluated. The body weights were 321g for Group C; 237g for Group Et; 333g for Group EE; and 278g for Group EtEE. Therefore, it was observed that Group Et had a weight 27% and 31% lower when compared to Groups C and EE, respectively. On the other hand, Group EtEE had weights 16% and 17% lower when compared to Groups C and EE, respectively, so that this was 11% higher when compared to Group Et, as per figure 1 (p<0.05).

**Figure 2:**
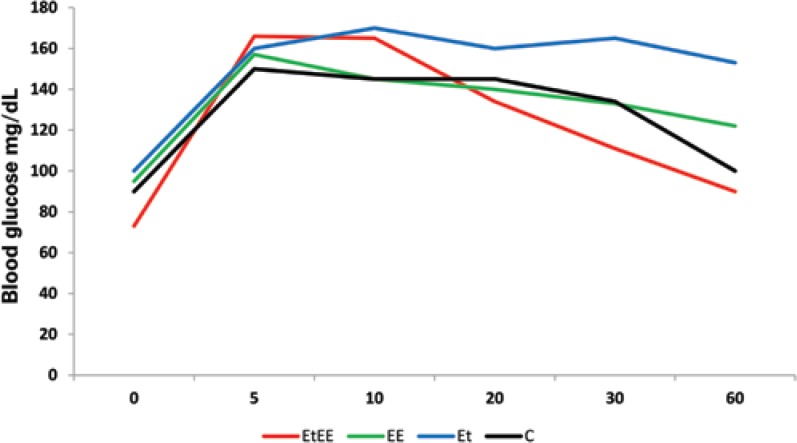
Representation of constant blood glucose drop in the groups: Control (C), Ethanol (Et), Electrostimulated (EE) and e Ethanol+Electrostimulated (EtEE)

As to blood glucose, Group C displayed a blood concentration of 100mg/dL; Et of 153mg/dL; EE of 122mg/dL; and EtEE of 90mg/dL. Thus, Group Et showed a 53% elevation in the first 60 minutes of electrostimulation compared to Group C. The blood glucose level of Group EE proved 22% higher than that of Group C in the first 60 minutes. As to Group EtEE, a 10% decrease in glucose concentration was noted when compared to Group C, as per figure 2.

**Figure 3:**
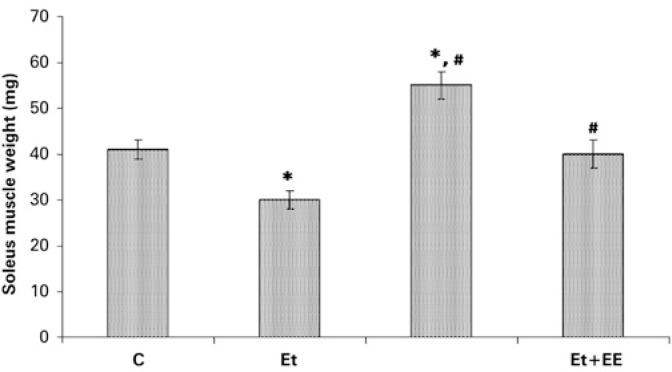
Soleus muscle weight in the groups Control (C), Ethanol (Et), Electrostimulated (EE) and Ethanol+Electrostimulated (EtEE). The values show mean±SEM, p<0.05 as compared to control (*) and to ethanol (#)

Regarding S muscle weight, it was evident that in Group C, the weight reached 41mg, in Group Et 30mg, in Group EE 54mg, and in Group EtEE 40mg. Therefore, Group Et showed a weight 25% lower than did Group C; Group EE showed values 34 and 83% higher when compared to those of Groups C and Et, respectively. Group EtEE showed no statistically significant difference relative to Group C, but had a weight 24% higher than did Group Et, as per figure 3.

Finally, the glycogen reserves of S and the entire gastrocnemius (GM) muscles were evaluated, as shown on table 1. As a result, the data in Group C was observed, which demonstrated 0.38mL of S and 0.41mL of GM. Whereas Group Et showed 0.26mL of S and 0.25mL of GM; Group EE had 0.57mL of S and 0.53 mL of GM; and Group EtEE showed 0.32mL of S and 0.30mL of GM. In this way, Group Et showed reserves 32% and 40% lower in the S and GM muscles, respectively, when compared to Group C. Group EE demonstrated higher reserves of S and GM when compared to C* (44 and 33%, respectively) and to Et (55 and 49%, respectively). Therefore, Group EtEE displayed glycogen reserves 16% and 25% lower in the S and GM muscles compared to Group C. The same group, compared to Group Et, demonstrated results 19% and 17% greater in the S and GM muscles, respectively.

**Table 1:** Glycogen reserves of the soleus and entire gastrocnemius muscles of the Groups Control, Ethanol, Electrostimulated, Ethanol+Electrostimulation

Groups	S	GM
C	0.38±0.03	0,41±0.01
Et	0.26±0.02*	0.25±0.03*
EE	0.57±0.03*^,#^	0.53±0.03*^,#^
EtEE	0.32±0.02*^,#^	0.30±0.01*^,#^

The values correspond to mean ± SEM compared to Control (*) and to Ethanol (#)

S: soleus muscle; GM: entire gastrocnemius; C: Control group; Et: Ethanol group; EE: Electrostimulated group; EtEE: Ethanol+Electrostimulated group.

## DISCUSSION

As to the evils caused by ethanol in the various organic systems, this study evidenced the intolerance to glucose absorption by the body and the reduction of muscular glycogen reserves, as well as the consequent decrease in the S muscle with the use of ethanol, which corroborates the findings in literature cited throughout this study.

As to the higher indices of blood sugar found in this study, we know that in the groups associated with ethanol (Et and EtEE), excessive consumption of this substance proved to have a direct relation with glucose metabolism alterations, favoring increased risk factors for cardiovascular diseases and the development of type 2 diabetes mellitus. This decreases the peripheral capture of glucose because of degradation of receptors, resulting in a drop in conduction of the mobilization signal of the glucose carrier towards the cell membrane^(7)^.

This fact justifies the findings exposed in this study, with an observed blood glucose elevation in Groups Et and EtEE (associated with ethanol), since the absorption of glucose by the cells tends to be made in an ineffective manner, besides the existence of gluconeogenesis inhibition in these individuals^(7)^.

On the other hand, we faced the view that ethanol, when administered at a low dosage, is associated with increased sensitivity to insulin, and is therefore favorable to glycogenic absorption, affording a beneficial effect in decreasing risk factors for the development of cardiovascular diseases^(7)^.

As to the muscle alterations observed in this study, literature has shown that under the chronic use of alcohol, muscle tissue is in a catabolic condition since it generates hypotrophy, which culminates in early atrophy, thus favoring the degeneration of type IIA muscle fibers, excessive inflammatory infiltrate, and severe atrophy of type IIA muscle fibers, as well as a motor deficit of muscle fibers, leading to muscle atrophy^(8)^.

When this is added to the nutritional status of the alcoholic (in general, insulin intolerance reduces the energy matrix of the muscle, and consequently, there is less muscular action)^(7)^, it corroborates the results of this study as to muscle weight, since the groups associated with ethanol (Et and EtEE) showed decreased weight when compared to the other groups. Regarding muscle glycogen reserves, it is known that insulin intolerance or the hindered action of this hormone reduces the peripheral capture of glucose reaching the skeletal muscle, which is the primary site responsible for the disposition of glucose by means insulin, a fact that favors the reduction of muscle glycogen reserves and reduced muscle action^(4)^. This is in accordance with the findings of this study, by which ethanol discouraged insulin activity and exposed the reduction of muscle glycogen reserves, besides having indirectly favored the lower muscle weight in groups with some form of association with ethanol^(4)^.

In this way, by evidencing smaller glycogen reserves in the body of the muscle in the groups associated with the treatment with ethanol due to the lower energy disposition generated, this study exposes the deleterious conditions of the muscle in the condition of ethanol ingestion.

As to EENM, studies have shown that during the recovery period, its application can minimize the deleterious effects that occurred in the skeletal muscle, maintaining the levels of muscle glycogen equal to those of healthy groups. This is because the impulse generated by EENM guides the electric field lines in such a way that there is sudden inflow of ionic sodium into the membrane of the motor nerve, generating an action potential. This stimulus is conducted by the axon to the synaptic gap, thus mediating muscle contraction^(9,10)^.

In this way, this finding propels the results of this study in finding better results in Groups EtEE and EE relative to Group Etas to muscle glycogen reserves.

We also conclude that, although in the literature low doses of ethanol have a beneficial effect in the body, high doses have a harmful effect, as was addressed in this project.

As for electrostimulation and muscle weight, we know that the electrical current resource is recognized as an important technique for maintaining the baseline condition of the muscle or for retarding the decrease in muscle compromise in deleterious situations^(10)^.

Therefore, the electrostimulated groups associated or not with ethanol showed higher muscle weights, corroborating the findings described in the literature.

Considering the alterations caused by ethanol, the electrotherapeutic resource used in this study justifies the events observed, in noting improvement of muscle glycogen indices concomitant to the increase and maintenance of muscle weight of the groups that received electrostimulation, compared to the groups with some form of association with ethanol.

## CONCLUSION

We conclude that exposure to ethanol, in the various systems under study in this project, was capable of decreasing the levels of glycogen in the S muscle in groups that had an association with ethanol, in such a way that EENM favored the containment of organic disorders caused by ethanol in all the groups in which it was associated. Parallel to these findings, it is possible to determine the influence of an alcoholic diet associated with the reduction in muscle and body weight, increased levels of blood sugar, so that EENM minimized the harm caused by the ethanol.
